# Supplementary material for: The “Historical Materials BAG”: A New Facilitated Access to Synchrotron X-ray Diffraction Analyses for Cultural Heritage Materials at the European Synchrotron Radiation Facility
Source: Molecules. 2022 Mar 20;27(6):1997. doi: 10.3390/molecules27061997 (PMC8950898; doi:10.3390/molecules27061997)
Supplement: Supplementary file 1 [file molecules-27-01997-s001.zip › molecules-1624631-supplementary.pdf]

Supporting Information

The "Historical Materials BAG": A New Facilitated Access to Synchrotron X-ray Diffraction Analyses for Cultural Heritage at the European Synchrotron Radiation Facility

1. X-ray Microscope and Sample Mounting at ID13

Figure S1 sketches the X-ray microscope at the ID13 micro-branch.

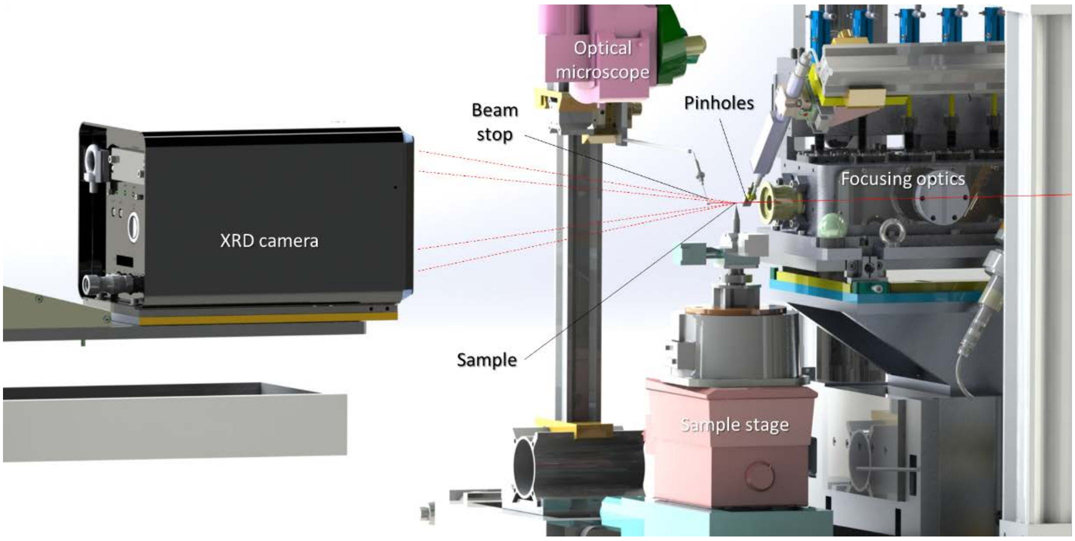

Figure S1. The ID13 X-ray microscope at the micro-branch. The X-ray beam is displayed as the solid red line, stopped by the beam stop. The dotted red lines represent XRPD signal. For readability, the XRF detector is not represented. It is mounted orthogonal to the beam, on the left side in the beam axis. (Thanks to L. Lardière, ID13, ESRF).

At ID13, the complete procedure for mounting and observing samples is the following:

- The XRPD camera, XRF detector and the beam stop are shifted away from the sample area.
- The sample stage is shifted downstream to avoid touching the last pinhole when removing and mounting sample holder.
- New samples are mounted and the sample stage is moved to its reference position.
- The optical microscope which stands downstream of the sample is moved down. Its focal plan has been tuned with the X-ray focus plane.
- The graphical user interface allows moving over the sample, adjusting the in axis position (optical focus) and defining regions and points of interest.
- When all acquisitions have been defined and stored in a queue, the optical microscope is moved up.
- The beam stop is moved at 2 mm from its reference position, letting the beam pass.
- A photodiode is inserted between the beam stop and the XRPD camera.
- An alignment procedure is started to align slits and pinholes, from the X-ray intensity collected on the photodiode.
- The beam stop is inserted in the beam and aligned.

- The photodiode is removed and the XRPD camera and XRF detector are moved back to their reference position.

This entire protocol takes about 30 minutes. Compared with the acquisition of maps which takes few minutes, it is therefore essential to mount together as many samples as possible. For this purpose, custom multi-holes sample holders are used, as shown in Figure S2.

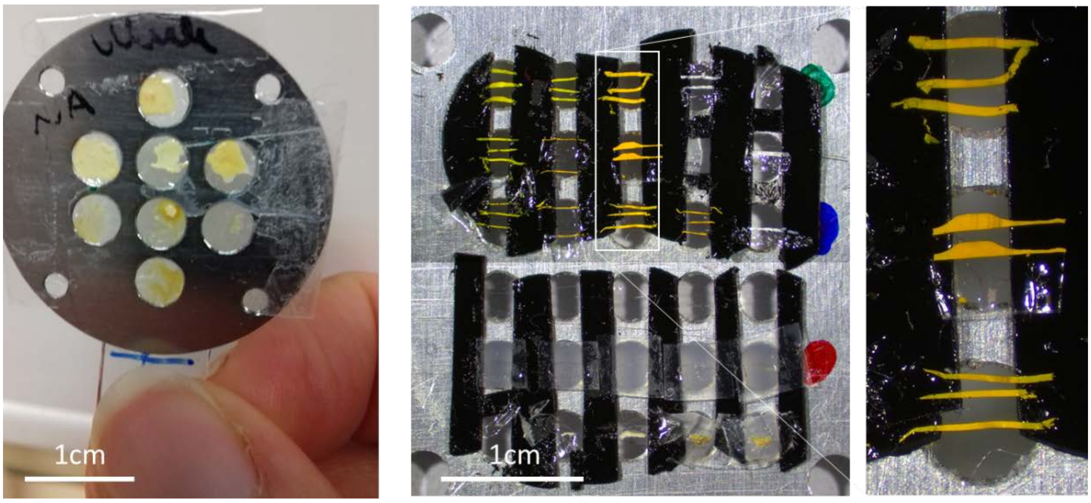

Figure S2. Two examples of sample holders at ID13. Scale bar in each picture represents 1cm. On the left, a 4mm diameter 8-holes holder with fragments of model paints. Samples are glued on tape. On the right, a 2mm diameter 30-holes holder with thin sections of model and historical paint samples. Sections are either pure paints (3 top rows) or painting fragments embedded in resin (3 bottom rows). Sections are glued on both edges with carbon tape, such that the XRPD signal comes from the paint only (no support material, see zoomed picture).

2. Daiquiri: The Graphical User Interface at ID13 and ID21

Once sample are mounted, navigation and selection of ROIs and POIs are done via the Daiquiri graphical user interface (Figure S3).

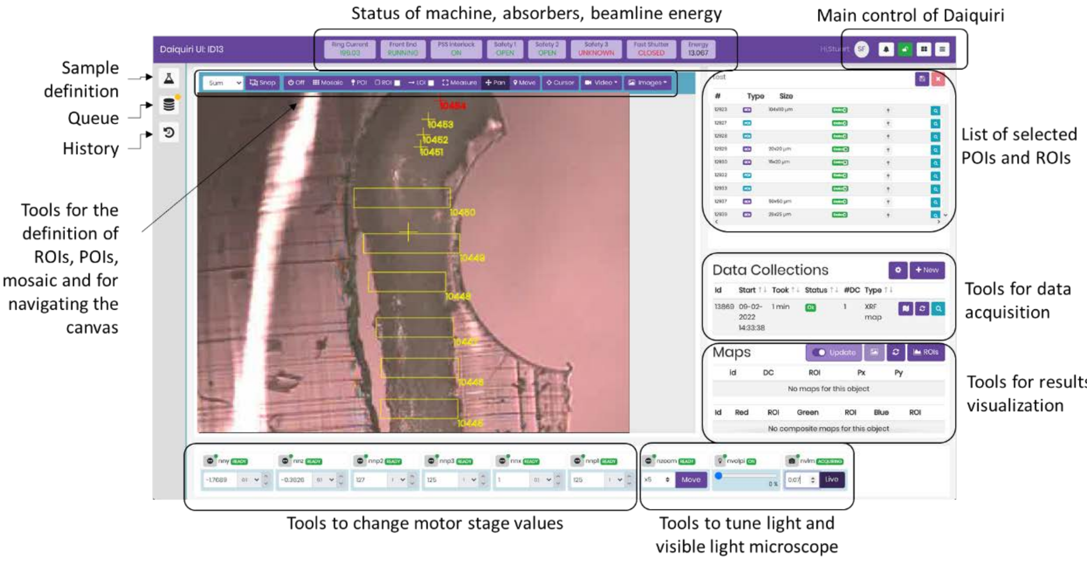

Figure S3. The Daiquiri graphical user interface and its main functionalities. The sample shown here is used for radiation damage studies. A series of maps and points were selected for the analyses detailed in Section 2.5.

3. Sample Preparation and ID21 FTIR Microscope for Radiation Damage Studies

The mock-up sample analysed in Figure 1 consists of lead white paint synthesized following the historical production method called the "Dutch stack process"; procedure described by Gettens et al. [1]. This paint was prepared with crude linseed oil from Linagro NV (Lichtervelde, Belgium) and was applied as a thin (~300µm) paint layer with a pigment-oil weight ratio of 4-1 on a polycarbonate substrate. After paint drying, thin sections were prepared with a rotary microtome. The sample preparation for this study was particularly challenging due to the technical constraints imposed by the combination of the two techniques (µFTIR/µXRPD). To meet the requirements of both techniques in terms of thickness, embedding medium and mounting, samples were prepared as thin sections ranging from 0.5 µm – 4 µm thickness without any embedding medium. The transmission signal was checked with µFTIR, after which the samples were mounted with tape on metal discs as self-standing structures (see Figure S2). This sample preparation method proved very effective and will be employed on similar samples in future experiments, since it efficiently eliminates undesired interferences of the embedding medium and of supporting materials and is suitable for both techniques.

The ID21 FTIR microscope is a Nicolet Continuum coupled to a Nexus Spectrometer. Spectra were collected in transmission mode, with a beam aperture of 15×15 µm² and using the internal Globar source.

4. XRPD Peak Shifting and Broadening during Radiation Damage Experiments

To evaluate the modification of XRPD patterns under prolonged exposure to X-rays, the XRPD peak area, position and FWHM were determined using the "Stack simple fitting" tool, in PyMca ROI Imaging. For this fit, a configuration file was created which includes a SNIP background and a Gaussian fit of the 12 most intense peaks in the 2-theta range 10-28° (area positive, position and FWHM constrained within 0.1°). For a better readability, results were smoothed over 100 consecutive scans and peak position values are shown relative to the initial peak position. Figure S4 displays the results for the (1 0 1), (1 0 4), (0 1 5) and (1 0 10) peaks. It shows that while the former has a rather stable width but shifting position, the three last peaks are stable in position, but broaden under the X-ray beam.

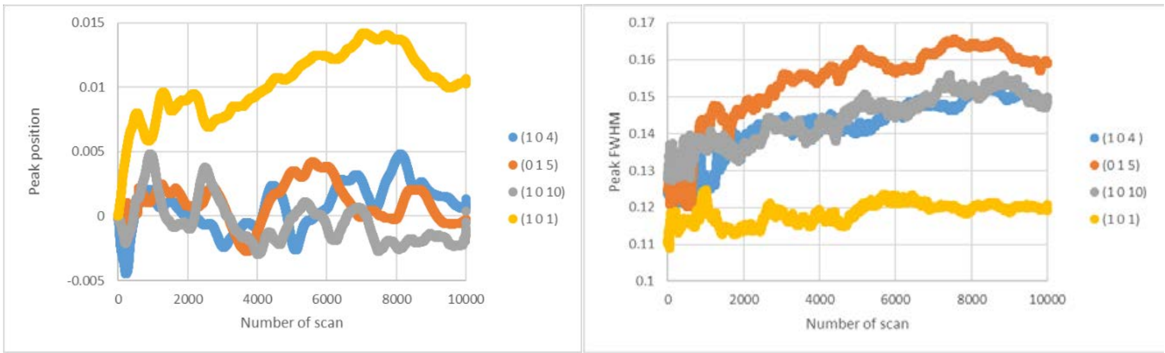

Figure S4. Evolution of peak position (left) and FWHM (right) of the (1 0 1), (1 0 4), (0 1 5) and (1 0 10) XRPD peaks in a lead white (mostly hydrocerussite) oil paint mock-up during repeated acquisitions of 10ms at low flux.

5. Radiation Damage on Chrome Yellow-Oil Model Paints

Similarly to the studies performed on lead white-oil model paints, additional analyses were carried out on chrome yellow oil model paints. Optical microscopy reveals a similar darkening of the paint (Figure S5a), while the formation of acids by consumption of esters is detected with µFTIR (Figure S5b and c).

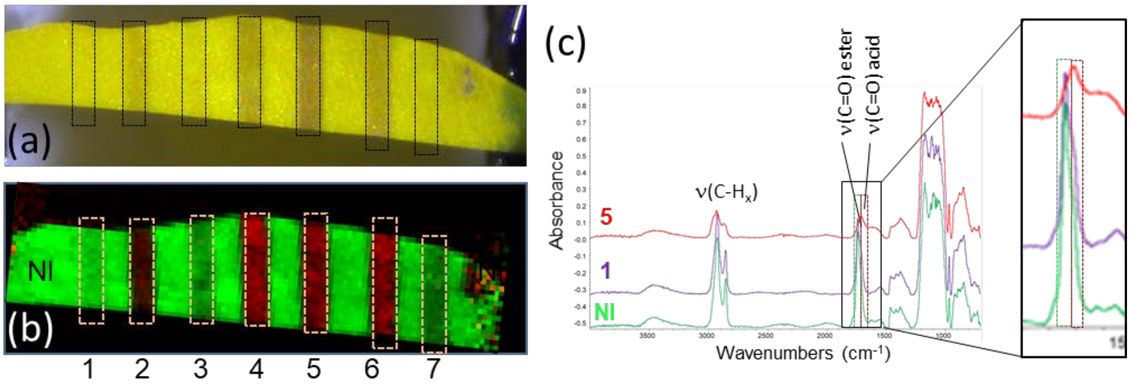

Figure S5. Assessment of radiation damage on a chrome yellow ($PbCr_{0.2}S_{0.8}O_4$) oil paint mock-up. Seven µXRPD maps were acquired at ID13, with the following conditions (1or 3 repeats; different dwell times; at low flux (LF) or high flux (HF)) : 1: 1 scan, 0.01 s, LF; 2: 1 scan, 0.01 s, HF; 3: 1 scan, 0.03 s, LF; 4: 1 scan, 0.03 s, HF; 5: 1 scan, 0.1 s, HF; 6: 3 scans, 0.01 s for each scan, HF; 7: 3 scans, 0.01 s for each scan, LF. The pixel size was 2.5×2.5 µm² to avoid overlap between two consecutive points. The map width was 50 µm and the height sufficient to cover the entire thickness of the paint sample. The maps are represented as rectangles in (a) and (b). (a) Optical microscopy after µXRPD. (b) µFTIR map acquired in transmission mode (beam size 15×15 µm², pixel size 10×10 µm², 50 cumulated scan per spectrum) after performing the µXRPD maps. The red/ green display shows the integrated intensity over the ν(CO) acid range (1674-1724 cm^{-1}) and ν(CO) ester range (1731-1759 cm^{-1}) respectively. These regions are displayed in (c) by a red and green rectangle, respectively. (c) Average FTIR spectra calculated over map 1, map 5 and a non-irradiated (NI) region.

6. ID21 X-ray Microscope

µXRF map shown in Figure 6b was acquired at the ID21 beamline [2]. The X-ray energy was set to 3.04 keV to have a good excitation of Mg. The coating of the primary mirror was chosen such to also have a third harmonic beam at 9.12 keV, to excite Ca and Fe as well. The beam was focused to 0.35 µm ver ×0.83 µm hor using a Kirkpatrick Baez mirror system. XRF was collected under vacuum, using a 100 mm² Sirius Series SDD detector from SGX Sensortech and a FALCONX4-2 Digital Pulse Processor from XIA LLC. XRF maps were batch fitted using the PyMca software.

References

1. Gettens, R. J.; Kühn, H.; Chase, W. T., 3. Lead white. *Studies in conservation* **1967,** 12, (4), 125-139.
2. Cotte, M.; Pouyet, E.; Salome, M.; Rivard, C.; De Nolf, W.; Castillo-Michel, H.; Fabris, T.; Monico, L.; Janssens, K.; Wang, T.; Sciau, P.; Verger, L.; Cormier, L.; Dargaud, O.; Brun, E.; Bugnazet, D.; Fayard, B.; Hesse, B.; Pradas del Real, A. E.; Veronesi, G.; Langlois, J.; Balcar, N.; Vandenberghe, Y.; Sole, V. A.; Kieffer, J.; Barrett, R.; Cohen, C.; Cornu, C.; Baker, R.; Gagliardini, E.; Papillon, E.; Susini, J., The ID21 X-ray and infrared microscopy beamline at the ESRF: status and recent applications to artistic materials. *Journal of Analytical Atomic Spectrometry* **2017,** 32, 477-493.
